# Evaluation of a whole system approach to diet and healthy weight in the east of Scotland: Study protocol

**DOI:** 10.1371/journal.pone.0265667

**Published:** 2022-03-24

**Authors:** Gavin Breslin, Wendy Wills, Suzanne Bartington, Charis Bontoft, Olujoke Fakoya, Imogen Freethy, Jaime Garcia-Iglesias, Neil Howlett, Julia Jones, Reda Lebcir, Nigel Lloyd, Katie Newby, Nigel Smeeton, Adam P. Wagner, Amander Wellings, David Wellsted, Katherine Brown

**Affiliations:** 1 Bamford Centre for Mental Health and Wellbeing, School of Psychology, Ulster University, Coleraine, Northern Ireland; 2 School of Health and Social Work, University of Hertfordshire, Hatfield, United Kingdom; 3 National Institute for Health Research (NIHR) Applied Research Collaboration (ARC) East of England (EoE), Cambridge, United Kingdom; 4 Institute of Applied Health Research, University of Birmingham, Birmingham, United Kingdom; 5 Department of Psychology, Sport and Geography, School of Life and Medical Sciences, University of Hertfordshire, Hatfield, United Kingdom; 6 Hertfordshire Business School, University of Hertfordshire, Hatfield, United Kingdom; 7 Norwich Medical School, University of East Anglia, Norwich, United Kingdom; PLoS ONE, UNITED STATES

## Abstract

Obesity is a global epidemic affecting all age groups, populations and income levels across continents. The causes of obesity are complex and are routed in health behaviours, environmental factors, government policy and the cultural and built environment. Consequently, a Whole System Approach (WSA) which considers the many causes of obesity and shifts the focus away from individuals as points of intervention and puts an emphasis on understanding and improving the system in which people live in is required. This protocol describes a programme of research that will: critically evaluate the evidence for WSAs; assess longitudinally the implementation of a WSA to diet and healthy weight to explore the range of levers (drivers) and opportunities to influence relevant partnerships and interventions to target obesity in East Scotland. The programme consists of four workstreams within a mixed methods framework: 1) Systematic review of reviews of WSAs to diet and healthy weight; 2) Longitudinal qualitative process evaluation of implementing two WSAs in Scotland; 3) Quantitative and Qualitative momentary analysis evaluation of a WSA; and 4) the application of System Dynamics Modelling (SDM) methodology to two council areas in Scotland. A Public Involvement in Research group (PIRg) have informed each stage of the research process. The research programme’s breadth and its novel nature, mean that it will provide valuable findings for the increasing numbers who commission, deliver, support and evaluate WSAs to diet and healthy weight nationally and internationally.

## Introduction

Obesity is a global epidemic affecting all age groups, populations and income levels across continents. Traditional approaches to tackling obesity through policy and programme intervention have had limited impact in reversing obesity trends [1]. More recently, Whole System Approaches (WSAs) have been used to address diet and healthy weight, yet evidence for their effectiveness remains in its infancy [2]. A WSA can be described as a collection of integrated and comprehensive interventions that aim to change the community system by targeting individuals, groups and community-level environments and policies. A recent systematic review of 65 studies examining the implementation and effectiveness of WSAs, 33 of which were on obesity, showed improved health outcomes: reductions in body mass index (BMI); increased parental and community awareness; community capacity building; nutrition and physical activity environment changes; and improved safety and wellbeing of community members [2]. Success of a WSA was attributed to meaningful engagement of stakeholders and the community in making decisions, good governance, trust and capacity, sufficient time to build relationships, adequate finance, and the embedding of the WSA within broader policy. Although initial findings regarding WSAs are promising, a cautionary approach is advised as descriptions of what constitute a WSA and outcomes reported in some studies were limited. Furthermore, long-term evaluation of WSAs is lacking in the available literature and it remains unclear how WSA are implemented and how the interactions between the stakeholders, processes, and other elements driving the implementation process affect WSA success, an area where System Dynamics Modelling (SDM) can provide important insights [3]. To address some shortcomings of existing evidence regarding WSAs, Public Heath England (2019) [4] provided a six-phase framework, referred to as the ‘Leeds Beckett Model’ regarding how to apply a WSA to diet and healthy weight. The six phases include: 1. Set-up; 2. Building the local picture; 3. Mapping the local picture; 4. Action (i.e., selecting the interventions, approach, and who will tackle diet and healthy weight); 5. Managing the system network; and finally, 6. Reflect and refresh. To date, this model has been expanded to nine points by Public Health Reform (2019) [5], but a comparison between applying this framework against other frameworks or the modified Leeds Beckett model has not been conducted in Scotland or elsewhere, nor has a system using this model been mapped using SDM–a process that could help provide a richer understanding of all parts of the system and how they interact with each other leading to change. Evaluation of a series of WSA pilot projects in the East Region of Scotland offers an opportunity to investigate how WSAs are being implemented, how these change over time and what key factors lead to their effective set-up and delivery.

### Study aims and research questions

To describe a programme of research that will review the evidence for WSAs, assess longitudinally the implementation of a Whole System Approach (WSA) to diet and healthy weight to explore the range of levers (drivers) and opportunities to influence relevant partnerships and interventions to target diet and healthy weight in the east of Scotland.

1. What elements of a WSA model existed within two areas of East Scotland prior to the pilot project and what did this entail?
2. What is the current practice and how has it changed to either include the Leeds Beckett WSA framework, a modified Leeds Beckett model or incorporating an alternative WSA framework?
3. Which elements of the Leeds Beckett WSA have been implemented locally and what were the reasons for this?
4. How do the WSAs across Midlothian and West Lothian compare?
5. What impact did the implementation of a WSA have on: a) local stakeholders’ knowledge and understanding of how best to address diet and healthy weight issues; and b) local stakeholders’ service planning and delivery ethos, policy and practice?
6. What was the process of implementing the WSA and how was it experienced by local stakeholders?
7. To what extent did the WSA meet the needs of stakeholders aims to meet community needs?
8. What were the barriers and enablers to implementing a WSA?
9. To what extent was any implementation of a WSA sustained over time?
10. What factors enabled the sustainability of the WSA?
11. Has adopting a WSA benefitted any other areas of working beyond healthy diet and weight?
12. What are the implications in terms of the resources used, and associated costs, for sustainability of the WSA? What are the:
  - most resource intensive activities resulting from the WSA?
  - associated costs of these resources, and who bears them?
13. Using System Dynamics Modelling causality mapping, what are the organizational elements and processes that are relevant and demonstrate potentially effective implementation of a WSA to diet and healthy weight?

## Materials and methods

Four workstreams have been developed to answer the research questions.

### Workstream 1: Systematic review of reviews of whole system approaches to diet and healthy weight

The aim will be to conduct a review of reviews to synthesise ways that Whole System Approaches (WSA) to diet and healthy weight have been implemented and evaluated nationally and internationally. The review will assess the various theoretical approaches or models used to implement the WSA. The following research questions will be addressed in the review: What models or theories have been used to implement whole systems approaches? How have whole systems approaches been evaluated to date? What evidence is there of the effectiveness of whole systems approaches? What has been the contribution of the public and/or service users in the development of whole systems approaches?

Systematic searches will be carried out using Scopus, PsycINFO (ProQuest), the Cochrane Library, and MEDLINE databases. Key search terms were devised with the research team with support from an information specialist librarian and these were reviewed by a member of the Public Involvement in Research Group (PIRg). Search terms will cover WSA in promoting diet and healthy weight, such as: diet, nutrition, malnutrition, eating habits, eating behaviour, healthy weight, obesity, overweight, underweight, body fat, body fat distribution, body mass, body size, body weight, BMI, adiposity, weight loss, weight gain, exercise, sedentary, physical activity, lifestyle, behaviour change, ‘‘whole system approach” and related terms such as system approach; systems-based; system(s) modelling; collaborative; joined up; cross-sector; multi-disciplinary; inter disciplinary; integrated; local; multi-faceted; multi agency; community wide; inter organisation, as well as approach; strategy; scheme; intervention and program. Databases will be searched from 1995 to 2022 using a combination of text and Medical Subject Headings (MeSH terms). Additionally, reference sections of identified articles will be searched for further relevant articles. Review papers will be included if they satisfy all the following eligibility criteria: 1) A review of any type that assesses WSAs; 2) Available in English; 3) Review focus is on the application of a WSA to diet and healthy weight in any age group; and 4) Reported the approach, theory or model used to implement a WSA.

Covidence software (www.covidence.org) will be used to support title and abstract screening, to import full-text papers, resolve conflicts and extract data. Focus will be placed on the models and theories that have been used to implement whole systems approaches, as well as the types of evaluations that have been undertaken on these approaches and whether there is evidence of their effectiveness. Data on contribution from the public and/or service users in the development of whole systems approaches will also be extracted and analysed.

### Workstream 2: Longitudinal qualitative process evaluation of implementing a WSA

A two groups x three time point qualitative longitudinal design will be used to assess the implementation of a WSA to diet and healthy weight. This workstream will assess research questions 1–11. Focus groups and/or interviews will be conducted at three phases: approximately 6, 12 and 18 months after initiation of a WSA with members of the WSA Core Working Groups (CWG) in two council areas in Scotland and the Wider Stakeholder Networks (WSN). The CWG are responsible for leading/facilitating the local WSA and WSN, and are the wider group of stakeholders who will participate in workshops and in the implementation of the WSA. Two council areas have been selected for evaluation: Mayfield and Easthouses in Midlothian, and Whitburn in West Lothian. This selection is driven by their contrasting choice of methodology in implementing the Leeds Beckett Model of a WSA. Both areas share similar population demographics. Mayfield/Easthouses and West Lothian are in the top 20% most deprived areas in Scotland and are both characterised by underlying poverty, income deprivation, as well as greater concentrations of older adults.

### Participants

Participants will be recruited from two groups: 1. WSA Core Working Group (n = 6 from each council area), and 2. the WSA Wider Stakeholder Network who attended training during WSA workshops (n = 25 from each council area). Participants will be emailed with an invitation to participate by completing the Participant Information Sheet and Consent Form via REDCap (Vanderbilt University 2021), a secure online platform.

### Data analysis

Focus group and interview data will be analysed using Framework Analysis (Ritchie and Spencer, 2004), a type of data analysis that offers a structured, systematic approach to summarising and analysing qualitative data. It has particular use where multiple researchers are involved in analysing qualitative data, and where large qualitative datasets need to be summarised [6]. Public and Patient Involvement (PPI) input will be sought in the data analysis process and the coding of the interview data in order to allow the inclusion of the public perspective in the development and implementation of the coding framework.

### Workstream 3: Momentary analysis

#### Participants and procedure

Participants from the WSA Core Working Group and the WSA Wider Stakeholder Network will, in addition to focus groups and/or interviews, be invited to complete monthly Momentary Analysis (MA) [7] surveys throughout the duration of the project to capture changes in the system as they occur (addressing mainly research question 12). MA questions record levels of agreement to a series of statements either on Likert scales (which will be coded one to five) or scale of agreement (giving a value of 0–100). Open-ended, qualitative responses are also collected.

#### Data analysis

Statistical analysis of this quantitative data will utilise descriptive statistic summaries by time point, visual plots of changes over time and descriptive comparisons between sites. Comparisons will be made for all questions between sites. Questions logging activities of relevance to the WSA and time spent delivering these activities will be analysed to understand the resources and associated costs of delivering a WSA. Descriptive statistics will be used to summarise the range of activities and the time devoted to them. An indicative cost of delivering these activities will be determined by multiplying time estimates by a suitable hourly rate of employment. Depending on data collected, we will consider comparing sites to explore indications of differences in resource use between WSA approaches. We will augment these health economic considerations with explorations of how the two sites have utilised funding given to each to establish and deliver a WSA.

### Workstream 4: System dynamics modelling

System Dynamics Modelling (SDM) [8] will be used to help understand the nature of the interactions between the organisational components and processes driving WSA implementation. A key element of SDM is mapping out the structure of the problem/area to be explored through a process of ‘group model building’ that involves stakeholders working together to build a group understanding of the multiple components influencing a complex problem (implementation of WSA) and the causality relationships between them. Here, by following the principles of group model building, mapping and qualitative model building will be used to understand what key stakeholders consider to be the best way of incorporating a WSA into practice addressing Research Questions: 6, 8, 10. Casual loop diagrams (CLDs) will be developed based on the understandings produced from the focus groups, interviews, and momentary analysis (conducted for WS2/WS3), publicly available documentation, and the analysis of other mapping work conducted by stakeholders during workshops. Where required, additional interviews with participants will be conducted specifically to inform our SDM work to supplement our understanding of cause-effect relationships between components.

CLD development will be conducted through a collaborative process involving ongoing consultation with stakeholders to verify our understandings and interpretations. In particular, CLDs will be used to describe the components needed to deliver a WSA to diet and healthy weight and provide a description of how these interact and the relative importance of each. By mapping relationships between the elements, a more explicit understanding of how things change over time may be revealed and will highlight the interdependent variables and levers that may accommodate greater and sustained behaviour change [9]. The produced CLDs will be explored to understand how the identified components interact with each other, which interactions appear to most influence the implementation process, and implications for improving the delivery of WSAs.

### Co-production, Patient and Public Involvement (PPI)

Co-production is a central tenet of the PHIRST and our evaluation plans. This evaluation will be co-produced by the PHIRST with Public Health Scotland/East Region team and other national and local partners and stakeholders, including recipients of the service or programmes, all working together to plan, design, deliver, analyse and disseminate the evaluation. We will routinely communicate and consult with these partner organisations and stakeholders, and in addition present proposals and updates to our Independent PHIRST Advisory Board (composed of relevant stakeholders in the field of public health and evaluations, which includes academics, third sector, governmental and public expertise) and our Scotland specific Project Advisory Group (similarly composed of key stakeholders but with membership more closely reflecting the subject and area of the evaluation). The feedback they provide will continue to shape key decisions within the research process including design, ethics and dissemination.

The PHIRST Public Involvement in Research group (PIRg), is an integral part of the research team and will be involved in all stages of the research process for this evaluation. The PIRg are represented at our WSA Advisory Group. They have contributed to key aspects of the design and methodology, and will support data analysis, dissemination, and implementation work. Their input will be complemented by local service user input. A group will be convened for this purpose, with the assistance of partner organisations in Scotland.

### Ethical considerations and declarations

Ethical approval has been received through the University of Hertfordshire Health, Science, Engineering & Technology Ethics Committee, approval number HSK/SF/UH/04686(1) on the 4^th^ November 2021.

### Status and timeline of the study

WS1 will be completed by May 2022, while WS2, WS3 and WS4 will be completed by March 2023.

## Discussion

This programme of research has several unique contributions incorporated within the four comprehensive workstreams and mixed methods approach. The study’s novelty is increased by the inclusion of a comparison of a WSA model (Leeds Beckett Model) to a modified Leeds Beckett model longitudinally. The application of momentary analysis and System Dynamics Modelling will also provide valuable findings for those who commission, deliver and design whole or complex system approaches and diet and healthy weight intervention programmes nationally and internationally. There will also be important learning for the implementation of WSAs to other public health concerns beyond obesity that may be of interest to other organisations considering adopting a WSA.

Dissemination will initially consist of academic peer-reviewed publications for each of the workstreams: 1) a review of reviews of WSAs to diet and healthy weight; 2) a protocol article for a longitudinal study of WSA to diet and healthy weight in Scotland; 3) an article reporting the findings of the process evaluation of a WSA to diet and healthy weight in Scotland; 4) A System Dynamic Modelling case study article presenting the system map, feedback loops and how local health authorities can effectively implement a WSA to diet and healthy weight; and 5) a reflective article on the extent of the application of PPI throughout the development and implementation of a WSA. Other outputs will be developed and shared with non-academic policy, professional, and public audiences, including local authorities, service users and community organisations. The final report will be completed by March 2023.
